# The Significance of Elective Specialty Posting in Improving Awareness of Plastic Surgery among Medical Students in India: A Survey

**DOI:** 10.1055/s-0045-1811567

**Published:** 2025-09-22

**Authors:** A.P. Premlal, Akshata Menedal, Anu Anto Kallerey, Priyavrata Rajasubramanya, N. Praveen, Mohamed Najeeb K, Roshjo Roshan Attokaren

**Affiliations:** 1 Department of Plastic and Reconstructive Surgery, Government Medical College, Thiruvananthapuram, Kerala, India; 2 Department of Plastic and Reconstructive Surgery, Government Medical College, Kozhikode, Kerala, India

**Keywords:** plastic surgery, focus group discussion, survey, perceptions, medical students

## Abstract

**Background:**

Plastic surgery, its broad range of procedures and scope, is as poorly understood by medical students as among the public. Mass media generated misconceptions result in plastic surgery being limited to cosmetic surgery in popular imagination, while in reality plastic surgery also encompasses hand and microvascular surgery, craniofacial, and burns among other domains. Correcting the perception of medical students is vital to ensure timely and optimal care for patients needing referral to a plastic surgeon.

**Objective:**

This article aims to study the awareness of plastic surgery among Bachelor of Medicine, Bachelor of Surgery (MBBS) students.

**Materials and Methods:**

A focus group discussion was conducted by the Department of Plastic and Reconstructive surgery, Government Medical College, Kozhikode, Kerala, India, for MBBS phase 4 students who had chosen plastic surgery as an elective posting for 2 weeks, during which they observed patient management at the operation theater, ward, and outpatient department. They also participated in a suturing skills laboratory session. A Google Form pertaining to the scope of plastic surgery was distributed among all years of MBBS students and their responses were collected. The student's participation was informed, voluntary, and confidential. Our study design was an online questionnaire after a focus group discussion; data was collected over 4 weeks. We included the eight students who had participated in the focus group discussion after completion of plastic surgery elective. Incomplete responses were excluded.

**Conclusion:**

There is a knowledge gap between perceptions of medical students and the realities of the scope and domains under plastic surgery. Students stated that they would benefit from a regular posting in their curriculum to enhance their understanding of plastic surgery.

## Introduction

Reconstructive procedures are known to have been performed since the 6th century BCE by Sushruta in ancient India. 1 Though the term plastic surgery derives from the Greek *plastos* “molded, formed,” 2 people often mistake it for the use of plastic/implants by interpreting it as “artificial.” 3 Public lack of awareness regarding plastic surgery seems to unfortunately be ubiquitous irrespective of time and place, whether it be in India, 2004, 4 Oman, 2020, 5 or Turkey, 2023. 6 This is further compounded by the fact that among the surgical subspecialties, plastic surgery is one that does not apparently refer to any organ system or procedures in its name, vis-à-vis other specialties such as cardiothoracic, genito-urinary, or neurosurgery. Plastic surgeons are perceived to be involved with cosmetic procedures than with hand or cancer surgery as per a study of 899 individuals conducted by Blacam et al 7 in 2014.

Bachelor of Medicine, Bachelor of Surgery (MBBS) students with their limited clinical exposure frequently have the same preconceived notions regarding plastic surgery as the general public. These ideas are fueled by media depictions of celebrities who have gone under the knife to enhance their appearance. This gets carried over to reflect even in qualified nonsurgical practitioners as illustrated by the survey conducted by Panse et al in 2012. 8

The students opted for elective specialty rotation as per the recommendations outlined by the National Medical Council (NMC). 9 A focus discussion group was conducted at the end of this period to assess the changes in participant perceptions of plastic surgery arising as a result of their 2-week posting. This was compared with the medical student population at large.

## Objective

This article studies the awareness of the scope of plastic surgery and as a career option among MBBS students.

## Materials and Methods

A focus group discussion lasting 1.5 hours was conducted by the Department of Plastic and Reconstructive surgery, Government Medical College, Kozhikode, Kerala, India, for the eight MBBS third professional year (part II) students who had chosen plastic surgery as an Block 2 elective posting for 2 weeks and allotment was done based on availability in accordance with the NMC guidelines. During the rotations they observed patient management at the operation theater, ward, and outpatient department. They also participated in a suturing skills laboratory session to learn basic techniques of suturing. After the focus group discussion, an online questionnaire as a Google Form pertaining to the scope of plastic surgery was distributed among all the MBBS students from our institute. Eight students who participated in the focused group discussion were excluded. The student's participation was informed, voluntary, and confidential.

## Results and Discussion

A total of 204 completed Google Form responses were obtained, similar to the survey conducted by Conyard et al in Queensland that included 234 students and by Kidd et al comprising 192 students. 10 11 Of the Google Form respondents, 31.9% were third year part I, 27.5% were first year, 26.5% were third year (part II), and 14.2% were second professional year, respectively.

The low participation trends noted in our study and those around the world needs further exploration to ascertain whether it reflects an apathy toward the field, lack of understanding about the scope of the field, and perceived specialized nature of the field.

**Fig. 1:**
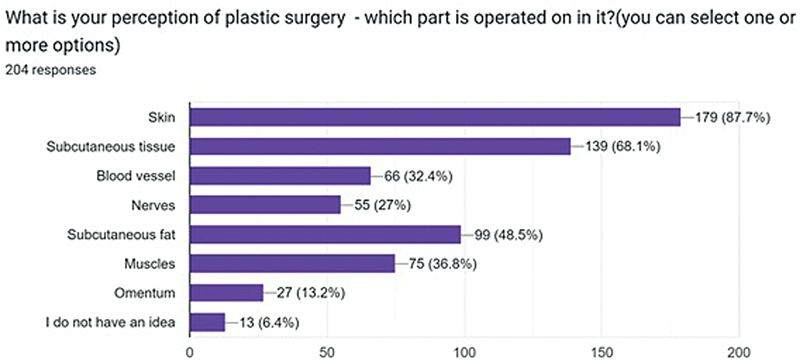
Perception of Plastic Surgery.

Our survey included the following questions, which also formed the points of discussion among the focus group members:

1. Perception of plastic surgery—the organ/tissues being operated on ( Fig. 1 )

During the focus group discussion, the majority of students associated it with cosmesis, enhancement of quality of life, and surgery performed on exposed parts. Most responses to the questionnaire also suggested that it involves surgery of skin only (87%) and subcutaneous tissue (68.1%). In a study conducted by Al Alawi et al in Oman even despite exponential advances in all the domains the public still underappreciates the field and poorly understands it too. 5

**Fig. 2:**
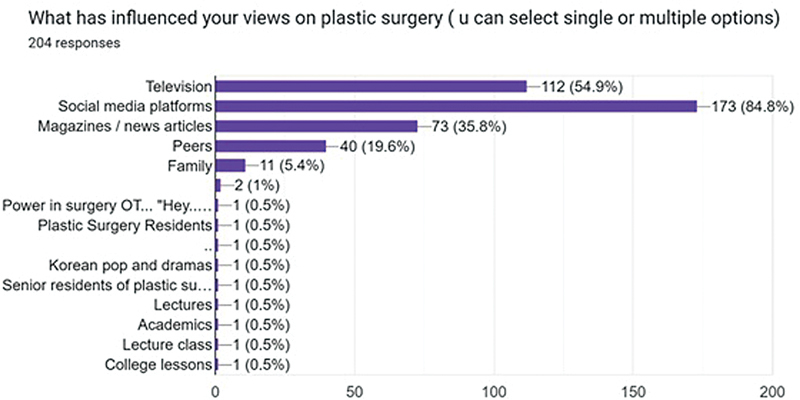
Major Influence on Perceptioning Plastic Surgery.

2. Major influences on perception of plastic surgery ( Fig. 2 )

The survey respondent's main influences were television (54%), social media (84%), magazines (35.8%), news articles and peers (19.6%), and family (5.4%). Less frequent responses were Korean TV shows, lectures, classes, academics, and plastic surgery residents. The focus group members elaborated on the impact of cinema and social media portrayal of “perfect” features to be their main influence. In a study conducted by Kidd et al, among medical students about the factors most influencing their perception of plastic surgery, majority responded placements within the specialty lecturers and medical school staff workshops/ courses. Other notably strong responses included role of media and medical school curriculum. 11

3. Impact of plastic surgery on patients' lives ( Table 1 )

**Table 1:** Additional questions from the survery

Survey questions	Responses in percentage		
	Yes	No	Not sure
Does plastic surgery have an impact on patient's life compared with other specialities?	87.30	1.90	10.8
Are cosmetic surgery and plastic surgery the same?	6.3	56.9	36.8
Does plastic surgery handle emergencies?	62.7	9.8	27.5
Is it useful to be included as a posting in your curriculum?	79.9	3.4	16.7

Both the focus group and the survey respondents felt that the field made an impact on patients' lives. The focus group members saw the workings of the department and concluded that it improves quality of life rather than life expectancy. They opined—that the public accepts poorer outcomes for a burn and trauma patients, but plastic surgeons help in restoring form and function.

4. Is plastic surgery limited to cosmetic surgery ( Table 1 )

The focus group understood that cosmetic surgery is only one of the aspects of plastic surgery after completion of the posting. Majority survey responses conveyed that they are not the same (56.9%), 36.8% were not sure, and a small minority considered it to be the same. A study conducted in Queensland by Conyard et al showed similar results as ours that medical students are not informed about the full scope of plastic surgery. Students are aware of the association of plastic surgery with cosmetic surgery but are unaware of the plastic surgeon's role in hand, craniofacial, and reconstructive surgery. To continue being recognized as specialists in hand, craniofacial, and reconstructive surgery, this gap between perception and reality needs to be addressed.

5. Role of plastic surgery in emergencies ( Table 1 )

The focus group shared that they believed plastic surgery to be a slow-paced field handling only minor, elective cases, but during their postings they realized the trouble shooting aspects of intraoperative calls of other specialties, handling varied spectrum of emergencies from scalp trauma to hand transplantation. In addition to routine ward rounds, elective operation theatre, and outpatient department. This differed from the survey responses—62.7% felt that plastic surgeons did handle emergencies whereas 9.8% responded in negation. Note that 27.5% were not sure about it.

**Fig. 3:**
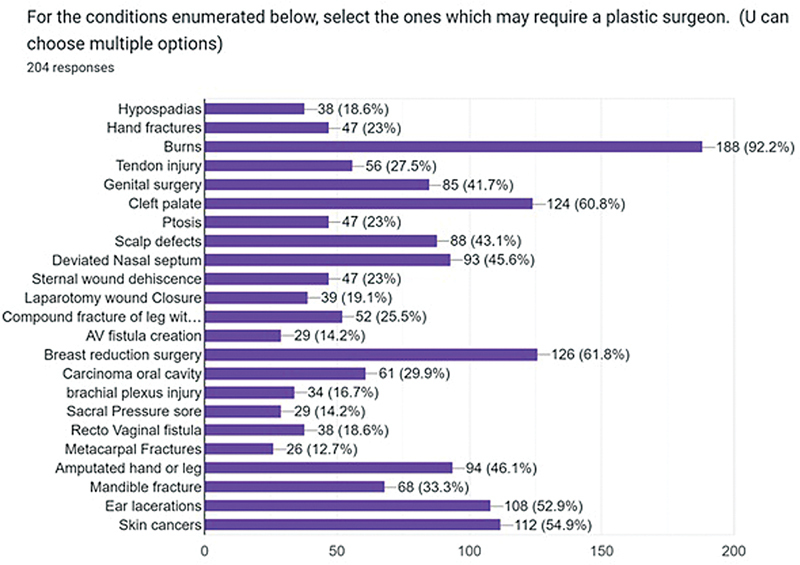
Condition primarily managed by a plastic surgeon.

6. Conditions primarily managed by a plastic surgeon ( Fig. 3 )

Fig. 3 depicts that maximum students thought burns, cleft palate, breast reduction, replantation of amputated extremity, ear lacerations, and skin cancers are treated by plastic surgeons. This extent of positive response might also be because of a bias among the students, as the procedures asked as a part of the study will definitely be related to plastic surgery. There was a shortcoming of not adding procedures in which plastic surgeons were not involved. However, questions from other studies conducting similar assessment, such as the one in South Africa by Rogers et al in 2013, of 33 students, were structured similarly with similar shortcomings. 10

7. Usefulness of a clinical posting in plastic surgery posting ( Table 1 )

All the focus group members and most questionnaire responses (79.9%) felt it was very useful and should be included in the clinical rotations. Additionally, the focus group members participated in a skills workshop for basics of suturing. They felt better taught in small groups, opportunity to acquire basic competencies of wound suturing on simulation props boosted confidence for subsequent internship.

The focus group discussion highlighted two most prominent gaps with respect to knowledge and attitudes:

1. The organs operated on/procedures performed.
2. Role of plastic surgeons in emergent/urgent conditions.

Various other considerations are noted in other similar studies around the world. A survey of 5,135 individuals among general public by Shah et al in the United States revealed several causes for confusion regarding plastic surgeons like problematic medical marketing and billboards among them. 13

An online questionnaire by Alyahya et al in 2021, also drew similar conclusions while revealing that social media was the source of most medical students' perceptions of plastic surgery than medical sources. They observed that senior students would have had personal/clinical experience and elective courses in plastic surgery. 14

This knowledge gap extended to qualified medical professionals as well. A 2012 survey of 100 nonsurgical specialists by Panse et al revealed that more than half of them were unable to correctly identify the cases that required plastic surgical intervention. 8

Focus group discussions are an established method of qualitative data collection that takes advantage of group dynamics. Formed of 6 to 12 individuals ensuring everyone's maximum contribution to the discussion. The discussion lasts for 1 to 2 hours. 15 16 Focus group discussions have been used to assess student opinions and needs with regard to changes or additions to their curriculum. 17

The theoretical and practical curriculum needs to be modified with a view to educating the future generations of doctors on the vast scope of plastic surgery, for two reasons. Primarily, to ensure optimum care and timely referrals, and second, for undergraduate trainees to have a holistic perspective of plastic surgery so as to make an informed career choice.

The idea of including plastic surgery as a subject in the undergraduate medical curriculum is not a novel idea. A study by Farid et al in 2017 consisted of a questionnaire administered to 243 students from the United Kingdom and Canada and concluded that students recognized the need for plastic surgery to be part of their curriculum, but differed on their preferred learning methods. 18

A 2021 survey by Fraser et al in Canada collected responses from 214 medical students about plastic surgery followed by two focus group discussions each comprising of eight participants to better understand the influence of media and to explore ways to counter this influence. 19

A 2016 electronic survey of medical students in Australia by Conyard et al observed that having completed a plastic surgery rotation improved their chances of correctly identifying the conditions that would be managed primarily by the plastic surgeons. 10

The representativeness of our group that participated in the discussion is severely diminished by the small size of the sample and purposive nature of sampling, thus precluding statistical analysis.

Going forward, we would like to statistically evaluate the effectiveness of short course programs of the nature conducted by British Plastic, Reconstructive and Aesthetic Surgery (BAPRAS) in 2010, in creating awareness among medical students. Davis et al organized surveys of those students both before and after the course and compared them with those of nonmedical students who did not attend the course. The awareness of medical students was higher than nonmedical students to begin with, but improved significantly in all the aspects studied after the course. The participants also expressed an increased interest in plastic surgery as an elective or a future career. 20

## Limitations and Future Direction

Only students who opted for plastic surgery as their Block 2 elective benefited from exposure to department activities and procedures. As the study was conducted by the department of plastic surgery, there is a bias inclining to favorable response.

Such surveys and discussions have leading nature of questions and lack blinding. However, since these issues were common in other similar studies corroborating our findings, we can consider our results comparable.

A follow-up study is needed to ascertain the impact of our survey and focus group discussion.

## Conclusion

There exists a pervasive gap in knowledge and attitudes toward plastic surgery among the medical student population. Toward alleviating this gap, we would recommend including an academic-cum-clinical rotation for MBBS students with a focus on (1) correct identification of the procedures performed primarily and conditions that would require referral or secondary involvement of a plastic surgeon, (2) career counseling—plastic surgery as a career, clinical rotation can provide students with an understanding of the specialty, and (3) engaging students in activities and workshops for skill acquisition.

## References

[1] Bath K Aggarwal S Sharma V Sushruta: father of plastic surgery in Benares J Med Biogr 2019 27 01 2 3 27885151 10.1177/0967772016643463

[2] Macionis V History of plastic surgery: art, philosophy, and rhinoplasty J Plast Reconstr Aesthet Surg 2018 71 07 1086 1092 29685841 10.1016/j.bjps.2018.03.001

[3] Overview of Plastic Surgery. Published March 22, 2021. Accessed September 3, 2023 at:https://www.hopkinsmedicine.org/health/treatment-tests-and-therapies/overview-of-plastic-surgery

[4] Agarwal P Perception of plastic surgery in the society Indian J Plast Surg 2004 37 02 110 114

[5] Al Alawi K Al Shaqsi S Al Hosni A Al Firaisi A Public perception of plastic and reconstructive surgery in the Sultanate of Oman: a crowd-sourcing national survey Eur J Plast Surg 2020 43 06 825 830

[6] Seyhan N Public perceptions of plastic surgery Turkish J Plast Surg 2023 31 02 49

[7] de Blacam C Kilmartin C McDermott C Kelly J Patient perceptions of plastic surgery J Plast Reconstr Aesthet Surg 2015 68 02 197 204 25455297 10.1016/j.bjps.2014.10.008

[8] Panse N Panse S Kulkarni P Dhongde R Sahasrabudhe P Awareness and perception of plastic surgery among healthcare professionals in Pune, India: do they really know what we do? Plast Surg Int 2012 2012 962169 22685647 10.1155/2012/962169 PMC3362827

[9] Electives-Module-20–05–2020.pdf. Accessed August 29, 2023 at:https://www.nmc.org.in/wp-content/uploads/2020/08/Electives-Module-20-05-2020.pdf

[10] Conyard C Schaefer N Williams D Beem H McDougall J The understanding of plastic and reconstructive surgery amongst Queensland medical students JPRAS Open 2016 8 14 18

[11] Kidd T Palaniappan S Kidd D Waterston S Attitudes, influences and perceptions towards plastic surgery amongst medical students JPRAS Open 2021 29 167 177 34258366 10.1016/j.jpra.2021.04.009 PMC8254079

[12] Rogers A D dos Passos G Hudson D A The scope of plastic surgery S Afr J Surg 2013 51 03 106 109 23941756 10.7196/sajs.1792

[13] Shah A Patel A Smetona J Rohrich R J Public perception of cosmetic surgeons versus plastic surgeons: increasing transparency to educate patients Plast Reconstr Surg 2017 139 02 544e 557e 10.1097/PRS.0000000000003020 28121896

[14] Alyahya T Zakaria O M Al Jabr F A Plastic and aesthetic surgery among medical students: a cross-sectional study SAGE Open Med 2021 9 2.05031212110543E1473 10.1177/20503121211054373 PMC8558811 34733513

[15] Kitzinger J The methodology of focus groups: the importance of interaction between research participants Sociol Health Illn 1994 16 01 103 121

[16] Freeman T ‘Best practice’ in focus group research: making sense of different views J Adv Nurs 2006 56 05 491 497 17078825 10.1111/j.1365-2648.2006.04043.x

[17] Lie D Shapiro J Pardee S Najm W A focus group study of medical students' views of an integrated complementary and alternative medicine (CAM) curriculum: students teaching teachers Med Educ Online 2008 13 03 1 13 10.3885/meo.2008.Res00252 PMC2759094 19823690

[18] Farid M Vaughan R Thomas S Plastic surgery inclusion in the undergraduate medical curriculum: perception, challenges, and career choice-a comparative study Plast Surg Int 2017 2017 9.458741E6 10.1155/2017/9458741 PMC5463111 28630768

[19] Fraser S J Al Youha S Rasmussen P J Williams J G Medical student perception of plastic surgery and the impact of mainstream media Plast Surg (Oakv) 2017 25 01 48 53 29026812 10.1177/2292550317694844 PMC5626192

[20] Davis C R O'Donoghue J M McPhail J Green A R How to improve plastic surgery knowledge, skills and career interest in undergraduates in one day J Plast Reconstr Aesthet Surg 2010 63 10 1677 1681 19926544 10.1016/j.bjps.2009.10.023

